# Comparison among artificial intelligence-based age estimation from morphological analysis of the pubic symphysis versus experienced and novice practitioners using a new atlas for component labeling

**DOI:** 10.1007/s00414-025-03511-4

**Published:** 2025-06-04

**Authors:** Javier Irurita Olivares, Juan Carlos Gámez-Granados, Ángel Rubio Salvador, Ana García Reina, Emma Gutiérrez Pascual, Laura Castillo Jiménez, Sergio Damas Arroyo, Oscar Cordón García, Inmaculada Alemán Aguilera

**Affiliations:** 1 Department of Legal Medicine, Toxicology, and Physical Anthropology. School of Medicine, University of Granada, Granada, Spain; 2 Department of Electronic and Computer Engineering, University of Córdoba, Córdoba, Spain; 3 Institut Català de Paleoecologia Humana I Evolució Social (IPHES-CERCA), Zona Educacional 4, Campus Sescelades URV (Edifici W3), 43007 Tarragona, Spain; 4 Departament d’Història I Història de L’Art, Universitat Rovira I Virgili (URV), Avinguda Catalunya, 35, 43002 Tarragona, Spain; 5 Department of Software Engineering and Andalusian Research Institute in Data Science and Computational Intelligence (DaSCI), University of Granada, 18071 Granada, Spain; 6 Department of Computer Science and Artificial Intelligence and Andalusian Research Institute in Data Science and Computational Intelligence (DaSCI), University of Granada, 18071 Granada, Spain

**Keywords:** Age-at-death estimation, Todd's method, Artificial intelligence, Component labeling, Traditional methodology

## Abstract

Traditional age estimation methods based on macroscopic observation has been criticized for being excessively dependent on the observer's experience. The aim of this technical note is to propose a new atlas to assist the forensic practitioner in labelling pubic symphysis components. Furthermore, intra- and inter-observer evaluation was conducted using both novice and experienced practitioners. Two experienced and two novice practitioners have used this atlas to label 1,127 identified pubes from autopsies. Furthermore, they have considered the phases of Todd's method (1920) to estimate the age of each pubis. A previously published, semi-automatic artificial intelligence rule-based method based on the C4.5 algorithm has also been used to recommend a specific age-at-death estimation from the human-defined labels, to be compared with the macroscopic age estimation performed by all observers. Linear weighted kappa coefficients indicate that the intra- and inter-observer error when using the new atlas is higher for novice practitioners (Kappa < 0,6) than for experienced practitioners (Kappa > 0,6). Component labeling produces less error than phase assignment following the traditional method only in the case of experienced practitioners. In addition, the artificial intelligence method achieves a global percentage of correct estimates similar to what the four practitioners can achieve. The proposed atlas can be thus considered an effective tool for component labeling. Besides, explainable machine learning techniques could help automate age estimation methods through component analysis. These techniques reduce subjectivity, but it is important that researchers engage in the process to ensure the replicability of the method. Nevertheless, these results must be regarded as preliminary until they are subjected to a more extensive evaluation by a larger cohort of observers.

## Introduction

The method proposed by Thomas Wingate Todd in 1920 [1], together with its extended Suchey-Brooks variant [2], remains one of the most frequently used skeleton-based age estimation methods in adults nowadays [3]. The limitations of both methods are well known and are common to most “traditional” phase-based methods (those based on the macroscopic analysis of the bone to assign a phase of a degenerative or developmental process): high intra- and inter-observer error rates and the need for extensive prior experience to correctly apply this type of visual methods, which goes beyond a brief prior training [4–6].

Numerous investigations have tried to propose new variants of Todd’s method with the aim of solving the said problems related to both precision and accuracy. For example, some works have developed specific standards for each population [7–12]. However, these population differences were probably not adequately justified [13–16]. Some studies achieve this aim by analyzing 2D and 3D images [17–21], or they try to reduce the estimation error by applying more robust analysis methods, such as geometric morphometrics [22] or the wide range of possibilities offered by explainable machine learning within artificial intelligence [23–26].

In a previous study [27], we proposed the automation of Todd’s method using explainable machine learning techniques in combination with expert knowledge. The application of component analysis and the C4.5 algorithm [28] in a semi-automatic method, where the age-at-death estimation is developed from a previous human labeling of the pubic symphysis traits, allowed us to obtain as a result a set of 34 rules easily interpretable by the forensic anthropologist. This proposal seems to overcome many of the limitations raised above, in addition to obtaining more accurate estimates than the original methods. Nevertheless, the accuracy of the results is contingent upon the efficacy of the component labeling process, which has yet to be validated. The original contribution only considered a single set of annotations of the pubic symphysis sample by an experienced practitioner. Hence, the uncertainty associated to the labeling process was not analyzed as in the current study. In addition, the effectiveness of the method for age-at-death estimation has yet to be subjected to a comprehensive evaluation, since the previous study only considered the use of automatic methods.

In summary, in 2022, we published in a specialized journal of computer science the methodology used to automate Todd's method using explainable artificial intelligence techniques [27]. The present technical note aims to evaluate the feasibility of this automation of Todd’s method from a forensic anthropology perspective. To this end, the objectives of this study are:

1. To present a new atlas, i.e. a textual and visual guide, which aims to facilitate the appropriate annotation of the morphological features of the pubic symphysis.
2. To evaluate the use of the new atlas for the identification of the morphological features considered in Todd’s and Suchey-Brook’s methods. The practitioners’ performance evaluation is based on analyzing the intra- and inter-observer error when considering this atlas, by both two experienced and two novice practitioners. This performance is also compared with the error made when phases are assigned directly following a macroscopic analysis (instead of considering components).
3. To analyze the accuracy in age-at-death estimation when using traditional phase-based methods, applied both by novice and experienced practitioners, when compared with a semi-automatic explainable machine learning technique for component analysis considering the application of the new atlas.

The results obtained will allow us to: i) analyze the influence of the forensic practitioner expertise in the pubic symphysis labeling process and help to systematize such process thanks to the proposed atlas; and ii) evaluate explainable machine learning for its practical application in forensic anthropology contexts, to determine if it can guide future lines of improvement for the development of new methods for estimating the biological profile. Nonetheless, it is acknowledged that the number of observers who participated in the study may be inadequate to substantiate reproducible results. Consequently, the conclusions obtained must be regarded as preliminary. It is imperative to include a larger number of participants in future studies to test the usefulness of the proposed atlas.

## Material and methods

The collection of identified pubes located in the Physical Anthropology Laboratory of the University of Granada (Spain) was used to conduct this study. This collection results from autopsy studies developed since 1991 and currently comprises pubes of 834 individuals. In most cases, the state of preservation is excellent and extensive *antemortem* information is available. The acquisition of this sample was the result of a collaboration agreement signed with the Granada Institute of Legal Medicine and Forensic Sciences.

To prepare the pubes for study, the largest amount of soft tissue was removed with the help of a scalpel, with great care so as not to alter the underlying bone. Subsequently, the pubes were cooked in water with sodium hexametaphosphate in an approximate proportion of 0,5 g/liter to induce degreasing of the bone and eliminate the remaining adhered soft tissues. They were then immersed in water for a period of approximately three months to eliminate any remaining soft tissue due to bacterial decomposition. Once this process was completed, a thin layer of vinyl acetate binder was applied to ensure its preservation. It is important to mention that this thin layer of binder is practically imperceptible and does not alter morphological characteristics of the pubis under study.

**Fig. 1:**
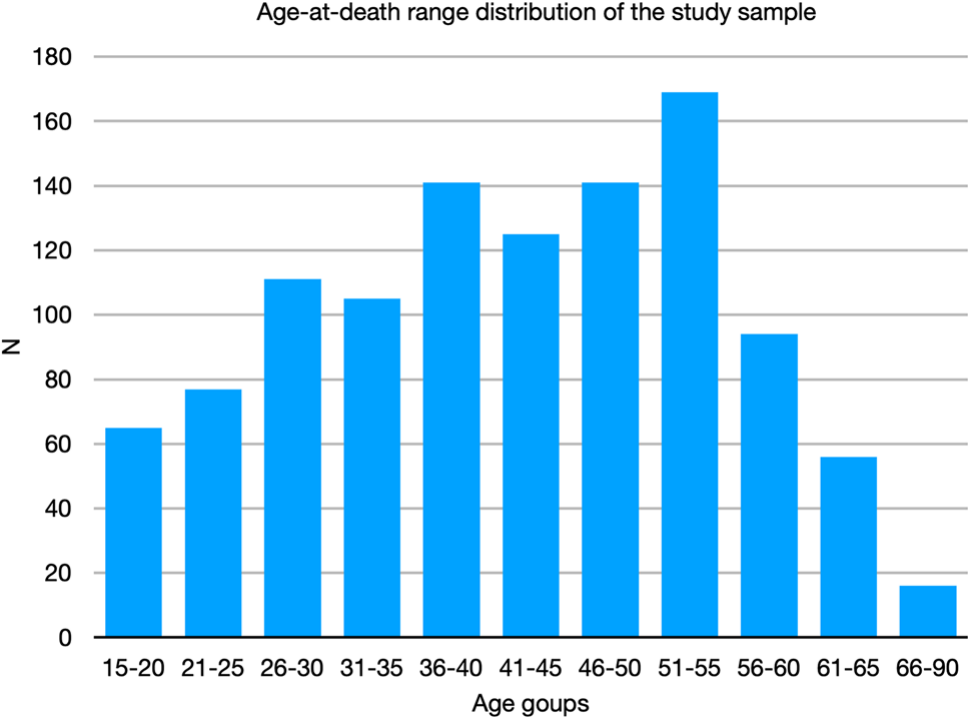
Distribution of the study sample by age-at-death groups

As a result, the pubes of 637 male and 197 female individuals are currently available, aged between 17 and 82 years and with abundant *ante mortem* information such as sex, age, country of birth, cause of death, possible pathologies, and sometimes additional information such as situations of alcoholism, drug dependence, obesity, anorexia, etc. In an effort to minimize the impact of extraneous variables on the findings of this preliminary study, only one sex and pubis without pathological or traumatic alterations have been used. So, the exclusion criteria have been poor state of conservation, non-availability or reliability of *ante mortem* information, and the presence of pathologies or traumatic alterations. Likewise, only the pubes of male individuals have been used. Finally, 1,127 pubic bones belonging to 566 individuals have formed the study sample. The distribution of the sample by age groups can be seen in Fig. 1.

**Fig. 2:**
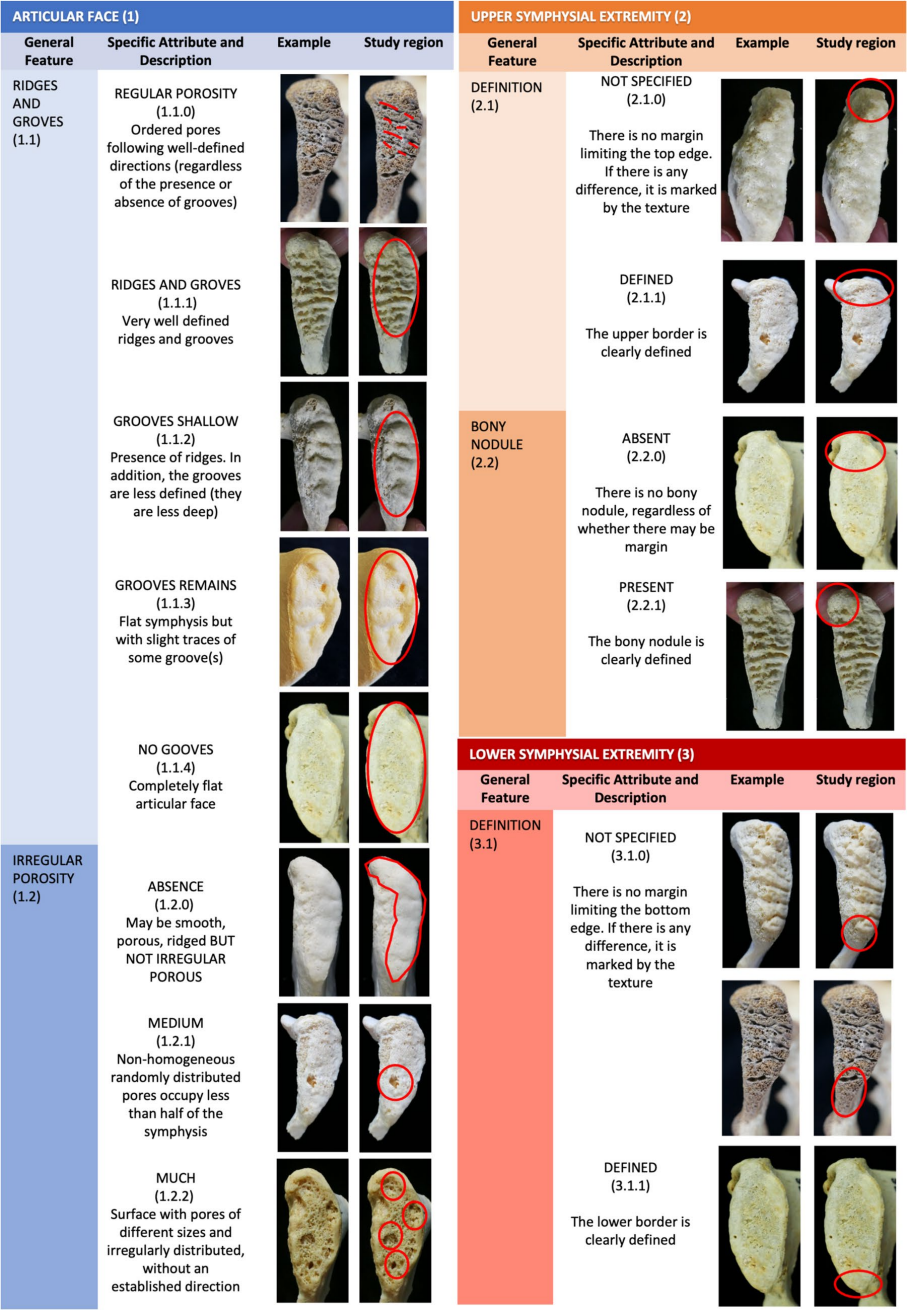
Atlas for component labeling: scoring system with descriptions of the pubic symphysis´ features and categorical values (part 1 of 2)

**Fig. 3:**
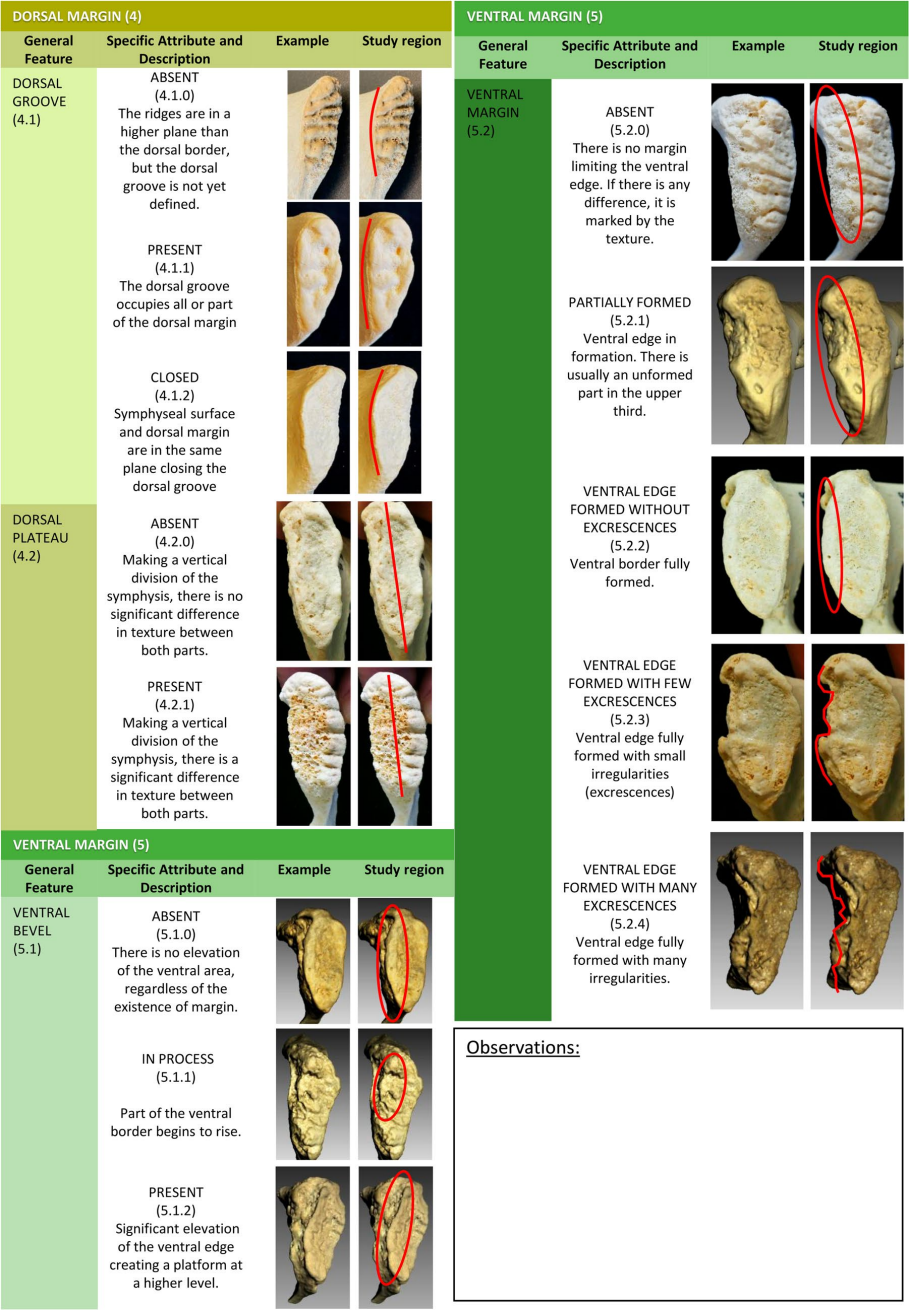
Atlas for component labeling: scoring system with descriptions of the pubic symphysis´ features and categorical values (part 2 of 2)

With the aim of facilitating the appropriate labeling of the morphological traits observed in the pubic symphysis, an illustrated atlas has been designed based on the criteria established by Todd [1] (Figs. 2 and 3). During the design process, we have also evaluated similar proposals of age-at-death estimation methods from the pubic symphysis [4, 26, 29]. We have selected the highest number of traits that can be easily identified to maximize the performance of artificial intelligence techniques. Likewise, we have identified as many levels of development/degeneration as possible for each of them. As a result, the detailed description of the 9 variables extracted from Todd’s descriptions [1], corresponding to pubic bone traits, have been incorporated into the atlas, each with a different number of levels depending on the complexity to describe and identify them. Definitions of each variable, prototypical images, and a numerical coding are included as a means to standardize the labeling process. Only one variable has been defined according to our own criteria which was not included in the original Todd’s descriptions: the dorsal groove. We propose this variable since, unlike the upper, lower and ventral margins, the dorsal margin originally proposed by Todd did not prove to be a useful variable in our study, as we visually observed it was always clearly delimited in the sample. Instead, the formation of a differential small groove was observed on the dorsal margin when the process of obliteration of the symphysis progresses. This grove subsequently disappears once the process is completed, and thus the trait becomes interesting and differential for estimating age-at-death.

The labeling process was carried out by four practitioners independently, based on the proposed atlas. Two of them were considered “experienced”, since they have more than 15 years of experience and thousands of skeletons analyzed in anthropological contexts. Despite having received sufficient training to apply age-at-death estimation methods, the other two practitioners were considered “novices” due to their status as students in their last year of their master's studies not yet having professional experience. After a prior training period to become familiar with the atlas, all the pubic bones were analyzed by the four observers. This process involved two tasks. First, the practitioner labeled each of the 9 components indicated in the atlas for each pubic symphysis. Then, (s)he was required to provide the global assignment of one of the 10 phases proposed by Todd for age estimation [1]. The choice of Todd’s method is due to two different reasons: i) the high number of phases proposed, and ii) the non-overlapping of the considered age-at-death intervals, which differs from later proposals [2]. Both aspects allow us to test the objectives set out in this study more efficiently. Subsequently, 160 randomly selected pubes were re-evaluated after a period of four weeks by both an experienced and a novice practitioner.

The artificial intelligence system for (semi-)automatic age-at-death estimation is based on the use of the C4.5 algorithm to automate Todd’s phase assignment process through the use of the component labeling of each practitioner, following a similar process to that shown in our previous study [27]. This algorithm is framed within the field of decision trees [28], one of the most used explainable machine learning techniques. In our case, its application makes it possible to derive a model based on a small number of simple and interpretable rules that can explain its decisions to the forensic scientists. Readers interested in details on the computational process to address model development can refer to [27].

We first analyze the intra- and inter-observer error assumed in two different tasks: i) the separate component analysis, and ii) the estimation of age at death by assigning one of Todd’s phases. This analysis is based on two different measures comparing the different decisions (trait labeling and age-at-death estimation) performed by the four forensic practitioners (Table 1): the linear weighted kappa coefficient [5] and the percentage of agreement between observations for each variable.

**Table 1:**
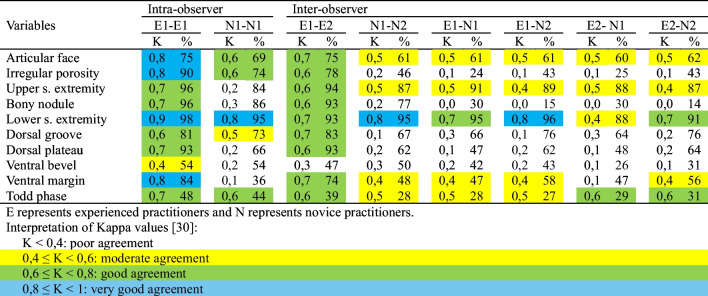
Intra- and inter-observer error with experienced and novice observers

**Table 2:**
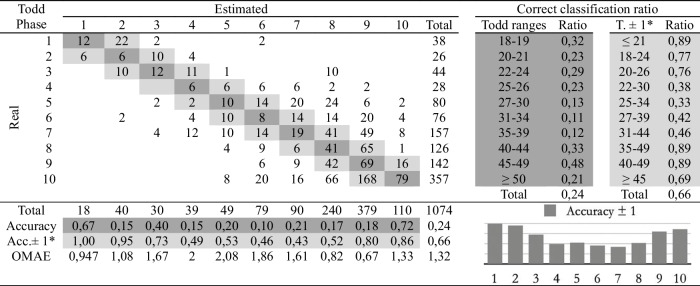
Confusion matrix of the macroscopic age assessment by experienced practitioner 1

*Adaptation of the ranges proposed by Todd. The phases before and after the estimated phase are considered correct

**Table 3:**
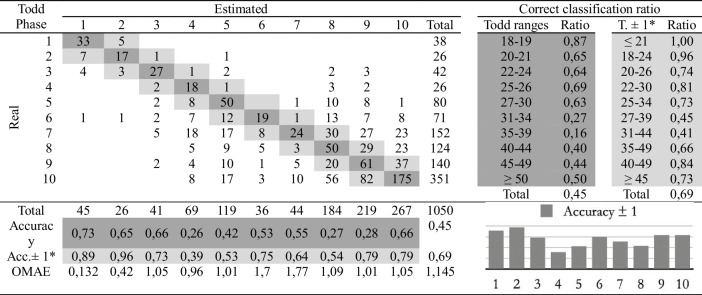
Confusion matrix of the automatic age assessment system through experienced practitioner 1 labelling

*Adaptation of the ranges proposed by Todd. The phases before and after the estimated phase are considered correct

**Table 4:**
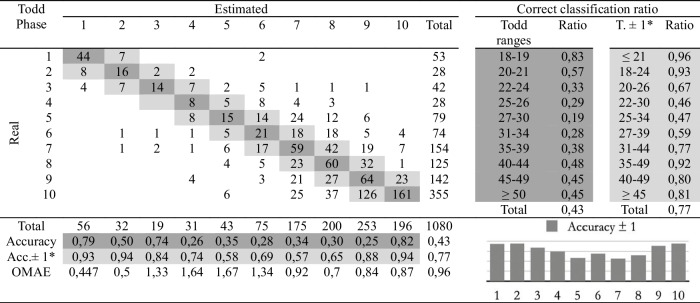
Confusion matrix of the macroscopic age assessment by experienced practitioner 2

*Adaptation of the ranges proposed by Todd. The phases before and after the estimated phase are considered correct

**Table 5:**
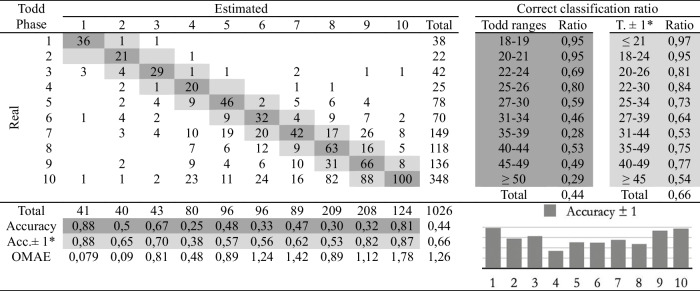
Confusion matrix of the automatic age assessment system through experienced practitioner 2 labelling

*Adaptation of the ranges proposed by Todd. The phases before and after the estimated phase are considered correct

**Table 6:**
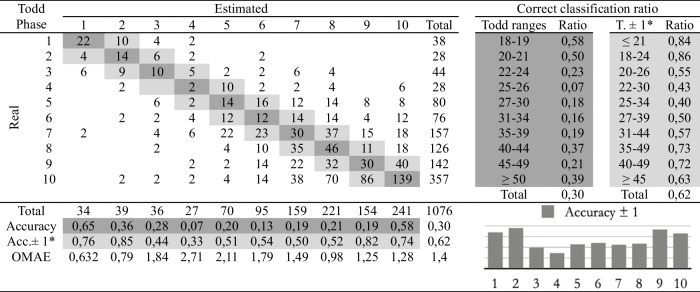
Confusion matrix of the macroscopic age assessment by novice practitioner 1

*Adaptation of the ranges proposed by Todd. The phases before and after the estimated phase are considered correct

**Table 7:**
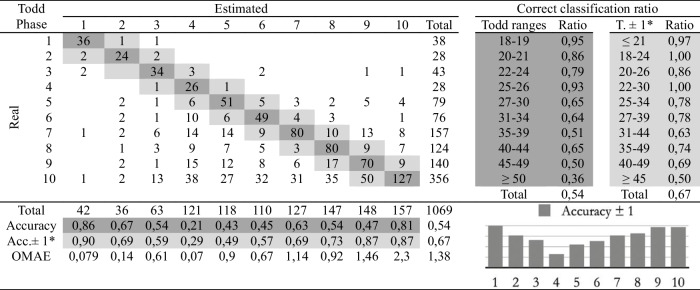
Confusion matrix of the automatic age assessment system through novice practitioner 1 labelling

* Adaptation of the ranges proposed by Todd. The phases before and after the estimated phase are considered correct

**Table 8:**
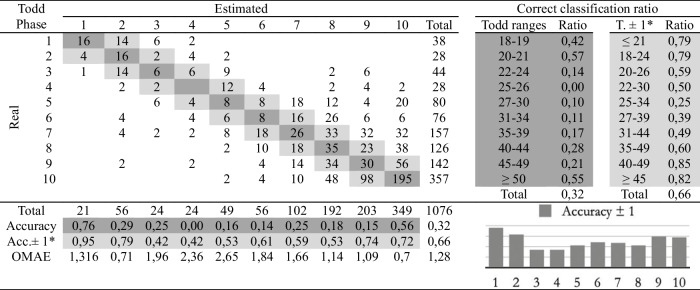
Confusion matrix of the macroscopic age assessment by novice practitioner 2

* Adaptation of the ranges proposed by Todd. The phases before and after the estimated phase are considered correct

**Table 9:**
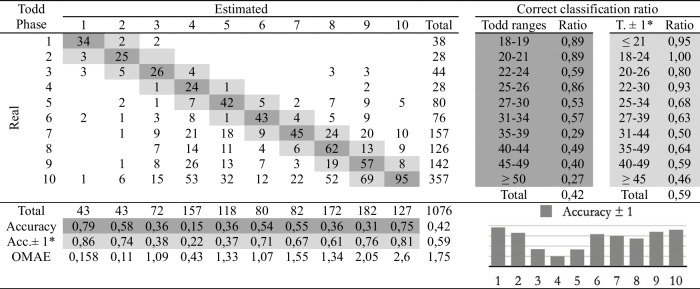
Confusion matrix of the automatic age assessment system through novice practitioner 2 labelling

* Adaptation of the ranges proposed by Todd. The phases before and after the estimated phase are considered correct

The second study involves comparing the estimations of age at death made by the four observers following the traditional methodology and the automatic method. Eight cross-tables have been developed to provide confusion matrices and estimation errors, two for each practitioner. The first table of the pair (Tables 2., 3., 4., and 5.), involves human macroscopic estimations, applying the standard Todd methodology to determine phases, excluding the use of the atlas-based component analysis. The second table includes the (semi-automatic) age estimations obtained by the machine learning method (Tables 6., 7., 8., and 9.). This method assigns a Todd phase and has been trained with the component labels assigned by the corresponding practitioner using the novel atlas. The AI method is applied to the pubes on which each expert scores the nine variables, while the experts estimate the decisions of those pubs and of some more that they do not fully label. Consequently, we should note that the total number of pubic bones analyzed in each table varies slightly, but the differences in number are very small (0 to 54 out of 1076). However, we consider that these minor discrepancies will not affect the conclusions reached in this study. Correct and incorrect estimates are indicated for each phase, and the following parameters were calculated:

- Correct classification ratio: ratio of individuals at each phase that have been correctly classified.
- Accuracy: rate of i-th phase instances correctly classified considering the actual instances in i-th phase.
- Ordinal Mean Absolute Error (OMAE): this parameter, measured in phases, allows us to measure the mean absolute error for ordinal classification problems, such as the current one.^1^It refers to the average deviation in absolute value between predicted and actual phase, thus defined in [0,Q-1], with Q being the number of Todd's age-at-death phases, i.e. Q = 10. Note that values close to zero indicate less error and therefore better classification.
- Adaptation of the ranges proposed by Todd with overlapping of one phase. Due to the high number of phases into which Todd divided the process, as well as the lack of overlap between the age intervals, an estimated phase before or after the chronological phase has also been considered a success in the estimate. Thus, we incorporate an overlap between the intervals of each phase and, according to the criteria of the practitioners of this study, we get closer to the real circumstances, where doubt between nearby phases is frequent.

## Results

Table 1 shows the results for intra- and inter-observer error when applying the new illustrated atlas and for the assignment of the phases proposed by Todd [1]. In order to interpret the linear weighted kappa analyses, the degrees of agreement offered by Ferrante and Cameriere [30] are used. These results show clear differences between novice and experienced practitioners, both for intra- and inter-observer error. The comparison between the two experts is good (Kappa ≥ 0,6) except for ventral bevel. However, when we carry out the same comparisons with the two novice practitioners, poor or moderate levels of agreement are obtained for most variables (Kappa < 0,6). Regardless of the observer’s experience, the variables that show greatest agreement are articular face and the definition of the upper and lower margins, indicating that these characteristics are less difficult to identify compared to the rest. The worst results are obtained with the ventral bevel variable. Finally, when experienced practitioners analyzed components separately, the corresponding kappa values and the percentage of coincidence are, in the majority of cases, better than when those experienced observers directly assigned phases.

Tables 2., 3., 4., 5., 6., 7., 8. and 9. show the confusion matrices related to the four observers. For each of them, two matrices are shown: one for the age-at-death estimates made macroscopically by the practitioners following Todd’s method [1] (Tables 2., 3., 4., and 5.), and another for the (semi-)automatic estimation using the artificial intelligence system trained on each human labeling of the components (Tables 6., 7., 8., and 9.). The most notable results are summarized below:

- The application of the age intervals proposed by Todd consistently yields higher overall accuracy values for the automatic method compared to the human practitioner, irrespective of the observer’s experience.
- When we consider an earlier and later phase as a success in the estimation, which is actually a realistic assumption due to the difficulty of the age estimation problem, the results obtained by the practitioners and by the automatic method are very similar. In almost all cases the accuracy is close to or even greater than 70%.
- In the former scenario, the practitioner who achieved the greatest number of correct estimates through macroscopic observation was expert 2 (Table 3.), with a 77% of correct estimates. This practitioner also outperformed the automatic method, which obtained a 66% of correct estimates (Table 7.), by a greater margin.
- The accuracy values for each phase show that the main limitation of all human practitioners, both experienced and novice, is the difficulty in macroscopically identifying the intermediate phases (phases 4 to 9). The machine learning method demonstrates a moderate capacity for identifying these intermediate phases, but only when non-overlapping Todd’s phases are considered.
- The lowest OMAE values are also those obtained by expert 2, with an average value of 0,96, compared to 1,26 obtained by the automatic algorithm. The interpretation of this data refers to the magnitude of the error in phases when the estimate was not correct. This means that estimations by expert 2 are always between the previous and the next phase of the actual phase in average.
- This fact is also easy to observe, in a general way, in the matrices of expert 2. In these matrices, the dispersion of the estimates is lower for the macroscopic method than for the automatic method. That is, when the algorithm makes mistakes, the range of those mistakes involves a greater number of phases.

## Discussion

The results of this study allow us to demonstrate and quantify the importance of the specialist’s experience for age estimation in forensic anthropology. High experience and expertise is essential for correct labeling of components. Thus, component-based methods are only more effective than phase-based methods when utilized by highly experienced practitioners. Methods based on explainable artificial intelligence techniques offer estimates that are comparable to those produced by humans, regardless of their experience. Consequently, they could provide a solution to the aforementioned issue. However, these methods are currently constrained by component labeling systems that are poorly replicable.

Table 1 shows that the difference between novice and experienced practitioners has been very notable. In the case of experienced practitioners, good agreement values have been obtained in practically all the variables analyzed (K ≥ 0,6), but the ventral bevel. However, poor or moderate agreement values have been obtained for almost all variables (K < 0,4) in the case of novice practitioners. The difference in performance between both groups indicates a high difficulty in identifying these traits, despite novice practitioners were previously trained and they were supported by the detailed atlas with definitions and prototypical images for each trait.

The results of the linear weighted kappa coefficients indicate that analyzing components individually instead of development phases in a macroscopic fashion only results in a moderate reduction in intra- and inter-observer error in the case of experienced practitioners. However, this is not the case for novice observers, who demonstrate greater consistency when analyzing phases rather than components. It is relatively straightforward to envisage a novice practitioner being able to distinguish between younger and older pubic bones after a few weeks of training. However, it is possible that they may require considerably more practice to become proficient in identifying variables such as macroporosity, bony nodules, and dorsal plateaus.

The findings of this study may call into question the conclusions previously drawn by numerous authors who claim that component-based systems are more objective and therefore offer more accurate estimates [4–6, 26, 31]. For example, Shirley et al. [5] compared the inter-observer error assumed when evaluating phases (with the Suchey and Brooks’ method) and when evaluating components. For this purpose, two expert observers evaluated 30 pubic bones of individuals aged 24 to 93 years. The results reveal a higher error rate in the evaluation of phases (linear weighted kappa of 0,68) compared to components (K ranked between 0,73 and 0,98). Although other prior studies do not make a direct comparison, as Shirley et al. [5] did, they have set out the merits of components over phases based on factors such as the extensive number of traits analyzed in each phase, their considerable variability, or the subjectivity in evaluating them [4, 6, 26, 31]. Nonetheless, as demonstrated by the outcomes of the current study, it is possible that this conclusion is exclusively applicable to practitioners with extensive experience. Consequently, incorporating novice practitioners is essential to accurately assess the replicability of new methods.

The high levels of agreement obtained to identify the formation of the upper and lower edges, both by experienced and novice practitioners, stand out positively. These results corroborate those obtained by most previous studies [4–6]. Despite the use of statistical analyses diverging from the one used in our study, these investigations substantiate that these traits exhibit low inter-observer variability. The upper and lower edges are easily identifiable pubic symphysis traits, with only two levels (see Fig. 2), that are fundamental for estimating age-at-death at an early age. The difficulty in assessing the changes that occur in the ventral bevel was also observed by other researchers [4, 31]. The definition of this variable is probably not clear enough or difficult to interpret, so our proposal for future studies is to change its definition or to eliminate it.

Finally, the new variable proposed for the analysis of the dorsal groove has shown good agreement among experienced practitioners (0,6 ≤ K < 0,8) but has also been difficult to identify for novice practitioners (K < 0,4). Despite this difficulty in identification, we consider that this variable represents an advance over the “dorsal margin formation” variable originally defined by Todd [1]. Shirley et al. [5] also analyzed the inter-observer error obtained when analyzing the dorsal margin according to the parameters defined by Brooks and Suchey [2], obtaining moderate agreement with this variable (K = 0,4–0,6). Other components related to the dorsal margin, such as the Lipping or the decomposition presented by other authors [26], are characteristic of older ages. Meanwhile, according to a preliminary assessment by the researchers of this study, the presence of the dorsal groove seems to be characteristic of individuals with intermediate ages (between approximately 30 and 40 years). The identification of distinct groups at intermediate ages represents a significant challenge for most age-at-death estimation methods. For instance, Brooks and Suchey [2] provide notably broad intervals for the intermediate phases; Schmitt et al*.* [8] also arrive at analogous conclusions. The use of contemporary AI-driven methods shows analogous patterns. Castillo et al*.* [26] achieve optimal outcomes with their proposed S4 model, which demonstrates superiority in identifying three distinct age groups: individuals below 30 years, those between 30 and 70 years, and those above 70 years. For this reason, future studies will be necessary to evaluate more precisely the usefulness of the dorsal groove for age-at-death estimation, particularly in intermediate age groups where lower accuracy has been currently reported to date [8].

Our study does not reflect a significant error reduction when we compare age-at-death estimation by phases following the macroscopic approach with methods based on component analysis (Tables 2., 3., 4., 5., 6., 7., 8. and 9.), results shared by other studies [4, 15, 26]. Even so, sometimes the estimation by phases following the schemes proposed by the traditional methodology can produce a smaller error than the estimation by components [31], as we observed in our study with experienced practitioner 2. It is our contention that the subjectivity of traditional methods as a defect and the ease of identifying components as a virtue are two insufficient criteria for declaring one approach better than the other. It should be noted that the effect of the observer’s experience is significantly more important using traditional phase-based methods, compared to component analysis. This is because the estimation is carried out from a holistic perspective considering all the variables globally. For instance, it is important to consider factors such as bone weight, which is strongly linked to processes associated with osteopenia and osteoporosity, as well as age [10].

In all disciplines, experience allows us to work more efficiently. Nevertheless, currently, experience is arguably the most crucial factor in forensic anthropology. Although reliable estimates can be derived when sufficient experience is available, demonstrating and replicating these results remains a significant challenge [32]. As stated by Schmitt et al*.* [8], the methods should give the same result regardless of the observer using them.

A preliminary examination of the findings presented in this technical note indicates that the utilization of the previously described rule-based explainable machine learning techniques for component analysis [27] may offer a potential solution. The automated algorithm estimates age-at-death with a similar degree of precision as that observed by the four practitioners through macroscopic assessment, regardless of their level of experience. In addition, as shown in [27], competitive global error values are also achieved in comparison with similar automatic methods proposed in other studies [17, 19, 20].

Meanwhile, as shown in Table 1, whether the estimation is performed by practitioners or by the artificial intelligence system, the main problem remains the component labeling process. As specified in [27], a rigorous validation process is followed to design the automatic rule-based method, which provides a clear understanding of the performance of the generated rule base. Nevertheless, as our findings illustrate, given the significant influence of the forensic practitioner’s experience on the labeling process, it can be postulated that the method is tailored to the observer analyzing the pubis sample.

It seems clear that artificial intelligence is a good alternative to improve methods for age estimation, although it is still necessary to improve the component labeling process. The following lines are proposed for future research:

- Promote the use of computer applications that facilitate a semi-automatic labeling process of characteristics and allow a more exhaustive training for the specialist through the use of numerous images and examples.
- Carry out the learning process of the algorithm from a component labeling data set obtained by the largest number of practitioners possible, avoiding “methods tailored to the observer”.
- Ensure that the algorithms used offer specific estimation errors for each age group.
- Ensure that automatic methods always give the same result regardless of who applies them. This will be a help for the observer, but not a substitute, since the observer’s experience will be what allows him/her to interpret the results, detect errors, and select the best methods for each case.

Although the number of observers considered is comparable to that employed in similar studies [5, 6, 11], the primary limitation of this study is that this is insufficient to justify the assertion of reproducible results. The process of labelling large samples of dry bones is inherently time-consuming. The labeling process of the pubis collection used in this study represents several months of dedicated work. Furthermore, it is required that observers work on-site, where the collection is available. In the case of professionals unable to work full-time on this task, the process can thus take years. To increase the number of observers in future studies, our team is developing specific software tools and implementing image analysis methods to streamline this task. This will allow a greater number of observers to analyze the sample without having to spend several months working at the bone site.

Finally, as a point of consideration for future studies, we intend to consider the high research potential of the Granada pubis collection, given the high amount of *ante mortem* information available. A study with similar aims to this one should be carried out with female subjects. In addition, the impact of traumatic or pathological changes on age estimation could be a valuable addition for future studies. These approaches could enhance the machine learning model’s capacity to interpret such cases effectively.

## Conclusions

- The new atlas for the identification of morphological features of the pubic symphysis has proven to be an effective tool for expert practitioners, but novice researchers still find it more difficult to identify the components than the phases.
- When the pubis is analyzed by experienced practitioners, all the traits considered, but the ventral bevel, can be properly identified. This does not occur in the case of novice practitioners, who have shown more difficulty in identifying the variables analyzed.
- Component analysis of the pubic symphysis allows reducing intra- and inter-observer error compared to phase analysis. However, this advantage is only achieved when the practitioner carrying out the study has acquired extensive experience.
- It is imperative that new methodological proposals include novice researchers, as methods designed exclusively for experts do not permit an accurate assessment of the replicability of the method in a realistic context.
- Component analysis allows the use of artificial intelligence techniques to design more objective and replicable methods, which globally achieve a percentage of accuracy in estimation like that achieved by macroscopic analysis, regardless of being designed from information (trait labeling) provided by either novice or experienced practitioners.
- An adequate labeling process is essential to design reliable component-based methods. Therefore, it is essential to analyze large samples by as many experts as possible to design methods based on consensus labeling.

## Data Availability

The dataset generated and analyzed during the current study is not publicly available due to privacy issues, but can be available from the corresponding author on reasonable request.

## Footnotes

1 The 10 age-at-death phases defined by Todd in his pioneering method are ordered according to the age range. Hence, the error made in estimating phase 5 (27–30 years) for an individual whose actual phase is phase 8 (39–44 years) is definitively greater than if phase 7 (35–39 years) is estimated. That is called an ordinal classification problem in machine learning and the OMAE provides an appropriate error measure for these kinds of problems.
